# Pharmacokinetics of Gepotidacin in Renal Impairment

**DOI:** 10.1002/cpdd.807

**Published:** 2020-05-19

**Authors:** Mohammad Hossain, Courtney Tiffany, Aparna Raychaudhuri, Dung Nguyen, Guoying Tai, Harry Alcorn, Richard A. Preston, Thomas Marbury, Etienne Dumont

**Affiliations:** ^1^ GlaxoSmithKline Collegeville Pennsylvania USA; ^2^ Currently CSL Behring King of Prussia Pennsylvania USA; ^3^ DaVita Clinical Research Minneapolis Minnesota USA; ^4^ University of Miami Division of Clinical Pharmacology Clinical and Translational Sciences Institute Wertheim College of Medicine, and Jackson Memorial Hospital Miami Florida USA; ^5^ Orlando Clinical Research Center Orlando Florida USA

**Keywords:** pharmacokinetics, physiologically based pharmacokinetic modeling, renal impairment, gepotidacin, safety, end‐stage renal disease

## Abstract

Gepotidacin is a novel triazaacenaphthylene bacterial topoisomerase inhibitor. In this phase 1, nonrandomized, open‐label, parallel‐group, multicenter, multipart study, the pharmacokinetics, safety, and tolerability of a single intravenous (IV) dose of gepotidacin 750 mg over 2 hours were evaluated in subjects with normal renal function, in those with moderate and severe renal impairment, and in end‐stage renal disease (ESRD) on and not on dialysis. Administration of IV gepotidacin 750 mg was safe and generally tolerated in the study subjects. Dosing in severe renal impairment with and without hemodialysis resulted in significant increases in plasma drug levels and decreases in clearance. The geometric mean elimination half‐life (t_½_) was minimally impacted (range 9.45 to 11.5 hours) in all the renal‐impairment groups relative to normal renal function. Regardless of renal function, urine gepotidacin concentrations remained considerably high over a 12‐hour period. Saliva concentrations displayed a linear relationship with plasma concentrations. The t_½_ in saliva was not impacted in the moderate‐impairment and ESRD subjects and was comparable to t_½_ in plasma. Over a 4‐hour dialysis, approximately 6% of the gepotidacin dose was removed. Overall, subjects with severe renal impairment and ESRD with and without hemodialysis may require adjustment in dose or dosing frequency.

Gepotidacin is a first‐in‐class triazaacenaphthylene bacterial type II topoisomerase inhibitor that inhibits bacterial DNA replication and has in vitro activity against susceptible and drug‐resistant pathogens associated with a range of conventional and biothreat infections.^1^, ^2^, ^3^, ^4^

Gepotidacin has demonstrated in vitro activity and in vivo efficacy against conventional and biothreat pathogens, including isolates resistant to existing classes of antimicrobials.^1^ Intravenous (IV) gepotidacin selectively inhibits bacterial DNA gyrase and topoisomerase by a unique mechanism that is not utilized by any currently approved human therapeutic agent. Structural data with a type II topoisomerase, DNA gyrase, reveals the novel binding mode of the class and distinguishes it from the binding mode of the quinolone antibacterials. As a consequence of its novel mode of action, gepotidacin is active in vitro against target pathogens carrying resistance determinants to established antibacterials, including fluoroquinolones.

In previous clinical trials with healthy volunteers, following IV administration of gepotidacin for single‐dose 1‐ or 2‐hour infusions at doses ranging from 200 to 1800 mg, peak blood concentrations of gepotidacin were achieved at the end of infusion with a subsequent rapid decline in drug concentrations due to a very short distribution half‐life. Geometric mean elimination half‐life (t_½_) values after a single IV dose were dose independent and ranged from 9.94 to 11.6 hours. Mean effective t_½_ values used to predict accumulation were also dose independent after repeat doses of 400 to 1000 mg twice‐daily, 2‐hour IV infusions (range 3.93 to 4.55 hours). Mean clearance (CL) values ranged from 30.0 to 44.7 L/h, and mean volume of distribution at steady state ranged from 105 to 196 L. In general, mean peak concentration (C_max_) and area under the concentration‐time curve (AUC) values for gepotidacin increased with an increase in dose. Following both oral and IV dosing of 1800 mg in the same subjects, the absolute bioavailability was 45%. Protein binding was determined in human plasma samples using equilibrium dialysis and was found to be low (33%).^7^

The results of an absorption, distribution, metabolism, and excretion (ADME) study for gepotidacin had indicated that, when administered as a single IV dose, approximately 60% of gepotidacin was recovered in urine. In addition, renal clearance (CL_r_) of gepotidacin was estimated to be approximately 40% of total systemic drug clearance.^4^ Therefore, renal impairment may have the potential to adversely affect the elimination of gepotidacin. This study was designed to describe the pharmacokinetics (PK) in renal impairment to allow for the development of appropriate dosing recommendations in patients with impaired renal function.

In a thorough QT study, infusion of gepotidacin at doses of 1000 mg and 1800 mg over 2 hours caused QTcF prolongation of 12 ms and 22 ms, respectively, at the end of the infusion, with a rapidly declining effect thereafter. Based on the exposure‐response (QTc) relationship, QT prolongation of approximately 13 ms (with an upper bound of the 90% CI of 15 ms) can be predicted at the highest achieved mean plasma levels in patients, around 9 μg/mL.^5^

Using PK data from neutropenic mice and data from a murine thigh infection model in which mice were infected with *S. aureus* or *S. pneumoniae*, the free‐drug plasma AUC/minimum inhibitory concentration (MIC) ratio was identified as the PK‐pharmacodynamic index most associated with the efficacy of gepotidacin.^6^

## Subjects and Methods

### Study Population

Eligibility criteria included male or female (nonpregnant, nonlactating) subjects between 18 and 80 years of age, inclusive. Part 1 included healthy subjects and subjects with moderate and severe impairment and end‐stage renal disease (ESRD) not on hemodialysis. Part 2 included subjects with ESRD on dialysis. Healthy subjects were in clinically stable health as determined by the investigator based on medical history, clinical laboratory results (serum chemistry, hematology, urinalysis, and serology), vital sign measurements, 12‐lead ECG results, and physical examination findings. Subjects with renal impairment had clinical laboratory values consistent with their disease. Normal renal function was defined as estimated glomerlular filtration rate (eGFR) ≥90 mL/min per 1.73 m^2^ or creatinine clearance (CLcr) ≥9 mL/min. Renal impairment criteria (part 1) included subjects with moderate renal impairment (eGFR 30 to <60 mL/min per 1.73 m^2^) and severe renal impairment with ESRD not on hemodialysis (eGFR <30 mL/min per 1.73 m^2^). eGFR was calculated using the isotope dilution mass spectrometry–traceable Modification of Diet in Renal Disease formula: eGFR (mL/min per 1.73 m^2^) = 175 × (serum creatinine) – 1.154 × age – 0.203 × (0.742 if female) × (1.212 if black). CLcr was estimated from a spot serum creatinine (mg/dL) determination: CLcr (mL/min) = [140 − age (years)] × [weight (kg) ÷ 72] × serum creatinine (mg/dL) (× 0.85 for female patients).

This study was conducted between June 2016 and June 2017 at 3 centers in the United States: Orlando Clinical Research Center (Orlando, Florida), DaVita Clinical Research (Minneapolis, Minnesota), and University of Miami, Division of Clinical Pharmacology (Miami, Florida) according to the ethical principles of “good clinical practice” and the Declaration of Helsinki after obtaining a written informed consent from each subject. The protocol and the informed consent were approved by IntegReview IRB (Austin, Texas) and Western Institutional Review Board (Puyallup, Washington).

### Study Design

This was a phase 1, nonrandomized, open‐label, parallel‐group, multicenter, 2‐part study that evaluated the PK, safety, and tolerability of a single IV dose of gepotidacin 750 mg over 2 hours. The study design was based on recommendations of the US Food and Drug Administration for studies in patients with impaired renal function.^8^ Because gepotidacin exhibits linear and time‐independent PK with no safety concerns with its metabolites, a single‐dose study was deemed adequate to achieve the study objectives.

Part 1 of the study included subjects with normal renal function, subjects with moderate and severe renal impairment, and subjects with ESRD not on dialysis. Part 2 included ESRD subjects on dialysis. Subjects with renal impairment were matched to subjects with normal renal function in terms of gender distribution, age (approximately ±10 years), and body mass index (approximately ±20%).

In part 1, subjects received a single dose of gepotidacin 750 mg administered as a 2‐hour IV infusion and remained in the clinic until all scheduled PK and safety assessments were completed up to approximately 48 hours after dosing. In addition, saliva samples (part 1 only) were collected for the PK analysis of gepotidacin concentrations. In order to proceed to part 2, in addition to the safety profile, the following PK criteria must have been met on review of part 1 data: observed mean values for AUC < 48 μg•h/mL and C_max_ < 14 μg/mL.

In part 2 of the study, ESRD subjects on hemodialysis participated in 2 treatment periods, with dosing in these periods separated by a washout period of at least 7 days. Subjects entered the clinic 1 day before dosing in each of the 2 study periods. In period 1 (day 1) subjects received a single dose of gepotidacin 750 mg as a 2‐hour IV infusion starting approximately 2 hours before the initiation of the last hemodialysis session of the week. In period 2 (day 8) subjects received the 2‐hour IV infusion of gepotidacin 750 mg starting within 2 hours after completion of the last hemodialysis session of the week.

### Dose Justification

The proposed single IV infusion dose of gepotidacin 750 mg administered over 2 hours was predicted to provide an approximate 2.5‐fold margin for mean C_max_ and AUC based on the highest single IV dose of gepotidacin (1800 mg) evaluated in adult healthy subjects.

### Physiologically Based PK Model Development and Qualification

A physiologically‐based pharmacokinetic (PBPK) model was developed for gepotidacin using a population‐based ADME simulator, Simcyp version 16 (Certara, Princeton, New Jersey), and developed using “middle‐out” approaches through integration of gepotidacin‐specific physicochemical properties, in vitro ADME drug‐dependent parameters, and human PK obtained after IV dosing of 1000 mg over 2 hours in a virtual white population of healthy adult subjects. The simulation input parameters are provided in Supplemental Table 1. Virtual healthy white and Japanese populations were used to verify and qualify the gepotidacin model against the PK results obtained from clinical studies.

Gepotidacin is eliminated via liver (unchanged gepotidacin represents 75% of plasma drug‐related material following IV administration) and kidneys via urinary excretion of the parent drug,^4^ with a calculated renal clearance of 16 L/h. In the in vitro phenotyping study, cytochrome P450 (CYP)3A4 was responsible for 99% of the observed oxidative metabolism. In addition, from a human radiolabeled mass balance study, unchanged gepotidacin and its metabolites accounted for 51% and 30% of total radioactivity, respectively, in the bile analysis following IV administration.^4^ Therefore, based on these findings, the fraction of the hepatic clearance carried out by the CYP3A4 was assigned as 0.25. CYP3A4 intrinsic clearance was derived using the retrograde calculator within Simcyp, based on the estimated hepatic clearance of 27 L/h (average total clinically observed IV clearance of 43 L/h and renal clearance of 16 L/h). An additional non‐CYP3A4 clearance was also incorporated into the model to capture the pathway of parent gepotidacin hepatic clearance. A full PBPK model setting was used for all simulations, and the volume of distribution of gepotidacin was calculated based on the permeability value of 1.5 × 10^‐6^ cm/s by method 2 in Simcyp. An additional perfusion‐limited organ was assigned with tissue:plasma partition coefficient of 100 to reflect the biphasic PK behavior observed in humans and supported by the rat quantitative whole‐body autoradiography data demonstrating that gepotidacin has high binding to pigmented tissues.

The PBPK model was verified by comparing simulated PK following single and repeat dosing with the available clinical data in healthy white adults. The verified model was then qualified in renally impaired populations for which gepotidacin PK data were available to confirm the predictive ability and robustness of the PBPK model. The simulations were performed with the assumption of only GFR changes in subjects with renal impairment and no active renal secretion.

### PK Assessments and Analyses

For all subjects, serial blood samples for PK analysis of gepotidacin were collected at the following time points: 0, 0.25, 0.5, 1, 1.5, 2, 2.5, 3, 4, 6, 8, 12, 24, 36, and 48 hours postdose.

Urine collection for subjects with normal renal function occurred at the following time intervals: predose, 0‐2, 4‐6, 6‐8, 8‐12, 12‐24, 24‐36, and 36‐48 hours. Urine collection intervals for subjects with renal impairment included predose, 0‐6, 6‐12, 12‐24, 24‐36, and 36‐48 hours.

Saliva samples were collected for PK analysis of gepotidacin in part 1 on days 1, 2, and 3 of dosing.

For ESRD subjects on hemodialysis, dialysate samples were collected only on day 1 of period 1 after dosing over each 1‐hour collection interval of the hemodialysis session.

Concentrations of gepotidacin were determined in plasma, urine, saliva, and dialysate samples using validated bioanalytical methodologies at PPD Laboratories (Middleton, Wisconsin). All samples were shipped frozen on dry ice and frozen at –20°C upon arrival.

#### Analyses of Plasma Samples

Blood samples were collected into K_2_EDTA tubes and immediately chilled on ice water. For analysis of plasma gepotidacin concentrations, a 25‐μL matrix aliquot of gepotidacin was fortified with 50 μL of 400 ng/mL gepotidacin‐d_7_ internal standard working solution in acetonitrile. Analytes were isolated through protein precipitation using acetonitrile and diluted with acetonitrile/mobile phase 10 mmol/L ammonium formate in water, pH 3 (1:1, v/v). The final extract was analyzed via ultrahigh‐pressure liquid chromatography–tandem mass spectroscopy detection using positive ion electrospray. The assay was validated over the gepotidacin concentration range of 10.0 ng/mL to 5000 ng/mL in human K_2_EDTA plasma.

Chromatographic separation of gepotidacin from other endogenous components was performed on LC 30‐AD from Shimadzu (Nexera; Kyoto, Japan). The analytical column was 50 mm × 2.1 mm, id 3.0 μm, Atlantis HILIC silica from Waters (Milford, Massachusetts), and the temperature was kept at room temperature. Mobile phase A was 10 mmol/L ammonium formate in water (pH 3), and mobile phase B was acetonitrile. The LC gradient profile was as follows: (1) linear gradient from 20% to 40% A from 0 to 0.8 minute, (2) isocratic profile at 40% A from 0.8 to 0.9 minute, (3) linear gradient from 40% to 20% A from 0.9 to 0.91 minute, and finally system equilibration at 20% A from 0.91 to 1.5 minutes acetonitrile:methanol (75:25, v/v), and mobile phase A was used as a needle wash for the sample manager. The flow rate was kept at 0.7 mL/min for the entire run. The typical injection volume was 4 μL.

The Sciex (Framingham, Massachusetts) tandem triple quadrupole mass spectrometer was used as detector in electrospray positive ionization mode. The following mass spectrometric parameters were used: ion spray voltage 2000 V, source temperature 550°C, collision energy 45 eV, declustering potential 76 eV, collision exit potential 20 eV, electrode potential 10 eV, curtain gas flow 25 psi, collision gas flow 10 psi, and mass resolution was set as unit. Quantification was carried out on multiple reaction monitoring of ion transitions of m/z 449.2 → 202 and 456.2→202 for gepotidacin and [^2^H_15_]‐gepotidacin, respectively. The bias and precision for gepotidacin were formally assessed in 3 core‐validation runs. The maximum within‐run percentage bias observed for any quality‐control level in any run was 8.41%, and maximum between‐run mean bias was 6.14%. The maximum within‐run precision was 7.96%, and the maximum between‐run precision was 5.41%.

#### Analyses of Urine, Saliva, and Dialysate Samples

A 25‐μL matrix aliquot was fortified with 25 μL of 50.0 μg/mL gepotidacin‐d_7_ internal standard working solution in acetonitrile. The samples were diluted with 1.45 mL of acetonitrile/mobile phase A (50:50, v/v) and mixed, and 30 μL was transferred to a new plate containing 0.97 mL of acetonitrile/mobile phase A (50:50, v/v) and mixed. The final extract was analyzed via high‐precision liquid chromatography with tandem mass spectrometry detection using positive ion electrospray.

Chromatographic separation of GSK2140944 from other endogenous components was performed on a series 1100 column (Agilent Technologies, Santa Clara, California). The analytical column was 50 mm × 2.1 mm, id 3.0 μm, Atlantis HILIC silica from Waters, and the temperature was kept at room temperature. Mobile phase A was 1 mol/L ammonium formate in water (pH 3), and mobile phase B was acetonitrile. The LC gradient profile was as follows: (1) linear gradient from 20% to 40% A from 0 to 0.8 minute, (2) isocratic profile at 40% A from 0.8 to 1.10 minutes, (3) linear gradient from 40% to 20% A from 1.10 to 1.20 minutes, and finally system equilibration at 20% A from 1.20 to 2.00 minutes. The flow rate was kept at 0.7  mL/min for the entire run. The typical injection volume was 10 μL.

The Sciex tandem triple quadrupole mass spectrometer was used as detector in electrospray positive ionization mode. The following mass spectrometric parameters were used: ion spray voltage 2000 V, source temperature of 550°C, collision energy 45 eV, declustering potential 76 eV, collision exit potential 20 eV, electrode potential 10 eV, curtain gas flow 6 psi, collision gas flow 12 psi, and mass resolution was set as unit. Quantification was carried out on a multiple reaction monitoring of ion transitions m/z 449.3 → 202.1 and 456.3→202.1 for gepotidacin and [^2^H_15_]‐gepotidacin, respectively.

The bias and precision for gepotidacin were formally assessed in 3 core‐validation runs. The maximum percentage within‐run bias observed for any quality‐control level in any run was 6.96%, and maximum between‐run mean bias was 4.37%. The maximum within‐run precision was 8.05%, and the maximum between‐run precision was 6.79%.

The urine assay was validated over the gepotidacin concentration range of 1.00 μg/mL to 500 μg /mL. The saliva assay was validated over the gepotidacin concentration range of 1.00 μg/mL to 1000 μg/mL. The dialysate assay was validated over the gepotidacin concentration range of 0.01 μg/mL to 5.00 μg/mL.

The in vitro protein binding of [^14^C]‐gepotidacin was determined in human plasma with various α_1_‐acid glycoprotein content using equilibrium dialysis methods.

### Safety Assessments

Safety assessments were conducted at baseline, during the dosing periods, and at the follow‐up visit and included the following: adverse events (AEs), clinical laboratory evaluations (chemistry, hematology, and urinalysis), pregnancy tests, vital signs, and 12‐lead ECGs. These data were descriptively analyzed.

### Statistical Analyses

PK parameters were estimated following a single IV dose of gepotidacin in subjects with mild, moderate, severe renal impairment, and in subjects with ESRD (on dialysis and not on dialysis) and compared with the PK parameters of matched subjects with normal renal function.

The sample size was considered sufficient to determine meaningful differences between the PK parameters in normal versus renally impaired subjects.^4^ The PK parameters were derived using standard noncompartmental methods using Phoenix WinNonlin Version 6.4 (Certara, Princeton, New Jersey). All PK end points were prospectively defined before analysis. Descriptive statistics were summarized for demographic variables. Plasma concentrations for all subjects, urine concentrations, and dialysate concentrations of gepotidacin and the associated PK parameters were summarized statistically (n, mean, median, SD, minimum, maximum, and coefficient of variation). Mean and individual plasma concentration‐versus‐time profiles were presented graphically on linear and semilogarithmic scales. The log‐transformed area under the concentration‐time curve from time 0 to infinity (AUC_0‐∞_), AUC_0‐t_, and C_max_ values for gepotidacin in the renal‐impairment groups and normal‐renal‐function groups were compared using an analysis of variance. Linear regression analysis was used to evaluate the relationships between estimated renal function and relevant PK parameters (eg, AUC, C_max_, and CL).

## Results

### Demographics and Disposition

Initially, enrollment included 8 subjects with moderate renal impairment and 8 subjects with severe renal impairment/ESRD not on hemodialysis. Subsequently, 8 subjects with normal renal function were enrolled who matched both the moderate and the severe renal impairment subjects. In total, part 1 enrolled a total of 24 subjects who completed the study as planned. In part 2, 8 subjects with ESRD on hemodialysis (matching subjects with normal renal function from part 1) were enrolled and completed the study as planned. Demographics and baseline characteristics of these subjects are summarized in Table 1.

**Table 1:** Summary of Demographics and Baseline Characteristics

	Part 1				Part 2
	Normal Renal Function	Moderate Renal Impairment	Severe/ESRD Not on Hemodialysis	Total	ESRD on Hemodialysis
Number of Subjects	(N = 8)	(N = 8)	(N = 8)	(N = 24)	(N = 8)
Completed, n (%)	8 (100.0)	8 (100.0)	8 (100.0)	24 (100.0)	8 (100.0)
Withdrawn, n (%)	0	0	0	0	0
Demographics/baseline characteristics					
Age (y), mean (SD)	61.5 (10.78)	72.0 (7.05)	61.4 (12.49)	65.0 (11.13)	51.8 (7.09)
Age ranges, n (%)					
Adult 18‐64 y	4 (50.0)	1 (12.5)	4 (50.0)	9 (37.5)	8 (100.0)
≥65‐84 y	4 (50.0)	7 (87.5)	4 (50.0)	15 (62.5)	0
Sex, n (%)					
Female	3 (37.5)	1 (12.5)	4 (50.0)	8 (33.3)	1 (12.5)
Male	5 (62.5)	7 (87.5)	4 (50.0)	16 (66.7)	7 (87.5)
BMI (kg/m^2^), mean (SD)	29.50 (3.929)	27.74 (4.383)	30.46 (6.175)	29.23 (4.846)	29.61 (4.786)
Height (cm), mean (SD)	169.85 (7.235)	169.76 (6.856)	165.31 (9.741)	168.31 (7.987)	177.96 (5.556)
Weight (kg), mean (SD)	84.99 (10.669)	79.99 (13.108)	82.60 (14.493)	82.53 (12.458)	93.50 (13.968)
Ethnicity, n (%)					
Hispanic or Latino	3 (37.5)	4 (50.0)	2 (25.0)	9 (37.5)	1 (12.5)
Not Hispanic or Latino	5 (62.5)	4 (50.0)	6 (75.0)	15 (62.5)	7 (87.5)
Race detail, n (%)					
African American/African heritage	1 (12.5)	1 (12.5)	2 (25.0)	4 (16.7)	8 (100.0)
American Indian or Alaska native	0	1 (12.5)	0	1 (4.2)	0
White, European heritage	7 (87.5)	6 (75.0)	5 (62.5)	18 (75.0)	0
Multiple	0	0	1 (12.5)	1 (4.2)	0
eGFR (mL/min per 1.73 m^2^), median (min, max)	114.2 (94, 155)^a^	43.3 (32, 54)	16.5 (8, 26)	NA	6.0 (4, 8)^b^
Childbearing potential, n (%)					
Postmenopausal	2 (25.0)	1 (12.5)	4 (50.0)	7 (29.2)	0
Sterile (of childbearing age)	1 (12.5)	0	0	1 (4.2)	0
Potentially able to bear children	0	0	0	0	1 (12.5)
Cardiovascular‐related medical conditions, n (%)					
Any condition	0	7 (87.5)	8 (100.0)	15 (62.5)	8 (100.0)
Angina pectoris	0	1 (12.5)	0	1 (4.2)	1 (12.5)
Diabetes mellitus	0	5 (62.5)	4 (50.0)	9 (37.5)	3 (37.5)
Hyperlipidemia	0	4 (50.0)	4 (50.0)	8 (33.3)	5 (62.5)
Hypertension	0	7 (87.5)	7 (87.5)	14 (58.3)	8 (100.0)
Myocardial Infarction	0	0	0	0	1 (12.5)

BMI indicates body mass index; eGFR, estimated glomerular filtration rate; ESRD, end‐stage renal disease.

a n = 5.

b n = 4.

### PBPK Modeling

Gepotidacin exposures simulated in healthy adult white subjects were compared with observed data for single doses of 400 mg to 1800 mg (Supplemental Table 2) and repeat doses of 1500 mg (Supplemental Table 3). Model‐simulated and clinically observed gepotidacin PK, both AUC_0‐t_ and C_max_, were in good agreement with <10% difference for all dosing regimens (Supplemental Tables 2 and 3). The outcome of these simulations confirmed the robustness of the PBPK model.

The model was validated for dose justification in renal impaired subjects (both moderate and severe renal impaired group). Gepotidacin exposures predicted in a population with normal (eGFR ≥ 90 mL/min per 1.73 m^2^), moderate (eGFR 30 to 59 mL/min per 1.73 m^2^), and severe renal impairment (eGFR < 30 mL/min per 1.73 m^2^) are provided in Table 2.

**Table 2:** Simulated Gepotidacin Pharmacokinetic Parameters in Healthy (Normal) and Renally Impaired Populations

Pharmacokinetic Parameters	Units	Healthy (Normal Renal Function) (GFR ≥ 90 mL/min)	Moderate Renal Impairment (GFR 30 to 60 mL/min)	Severe Renal Impairment (GFR 15 to <30 mL/min)
C_max_ (μg/mL)	Arithmetic mean (SD)	5.75 (1.04)	7.38 (1.40)	8.09 (1.63)
	Geometric mean (%CVb)	5.66 (18)	7.24 (19)	7.93 (20)
AUC_0‐t_ (μg⋅h/mL)	Arithmetic mean (SD)	17.9 (4.05)	26.6 (6.48)	29.9 (7.68)
	Geometric mean (%CVb)	17.5 (23)	25.8 (24)	29.0 (26)

AUC_0‐t_ indicates area under the concentration‐time curve from dose to last measurement; C_max_, maximum concentration; CVb, between‐subject coefficient of variation; GFR, glomerular filtration rate.

### Pharmacokinetics

#### PK in Plasma

Relative to normal renal function, the geometric mean plasma C_max_ of gepotidacin was elevated 1.2‐ and 1.6‐fold in subjects with moderate and severe renal impairment/ESRD not on hemodialysis, and 2.3‐ and 6.0‐fold in ESRD subjects before and after hemodialysis, respectively. Relative to normal renal function, the geometric mean plasma AUCs of gepotidacin were elevated 1.5‐ and 1.9‐fold in subjects with moderate and severe renal impairment/ESRD not on hemodialysis, respectively, and 2.4‐ and 4.1‐fold in ESRD subjects before and after hemodialysis, respectively. A summary of key PK parameters in plasma is provided in Table 3.

**Table 3:** Summary of Gepotidacin Plasma Pharmacokinetic Parameters by Renal Function

		Part 1			Part 2	
		Normal Renal Function	Moderate Renal Impairment	Severe/ESRD not on Hemodialysis	ESRD on Hemodialysis (Before Hemodialysis)	ESRD on Hemodialysis (After Hemodialysis)
Parameter	Units	(N = 8)	(N = 8)	(N = 8)	(N = 8)	(N = 8)
AUC_0‐∞_ (μg⋅h/mL)	AM	15.1 (3.19)	22.6 (3.86)	29.1 (6.09)	53.9 (75.0)	101 (119)
	GM	14.84 (20.7)	22.37 (17.0)	28.55 (21.4)	35.24 (95.0)	60.46 (140.5)
C_max_ (μg/mL)	AM	4.55 (0.733)	5.36 (0.833)	7.24 (1.50)	37.3 (87.1)	128 (184)
	GM	4.498 (16.3)	5.306 (16.0)	7.084 (23.3)	10.12 (216.3)	26.84 (797.1)
T_max_ (h)	Median (min, max)	2.00 (1.00, 2.00)	2.00 (1.50, 2.00)	2.00 (0.25, 2.50)	2.00 (0.50, 2.02)	1.58 (0.25, 2.00)
t_½_ (h)	AM	11.6 (1.34)	11.0 (1.51)	11.2 (1.50)	9.48 (0.806)	10.9 (1.72)
	GM	11.50 (11.9)	10.88 (16.1)	11.06 (13.4)	9.45 (8.4)	10.78 (16.4)
CL (L/h)	AM	51.5 (10.3)	34.0 (5.64)	26.8 (5.68)	25.4 (10.8)	17.9 (13.1)
	GM	50.55 (20.7)	33.53 (17.0)	26.27 (21.4)	21.28 (95.0)	12.40 (140.5)
V_ss_ (L)	AM	260 (55.9)	225 (32.4)	186 (49.2)	179 (85.4)	136 (117)
	GM	255 (21.7)	223 (14.8)	181 (23.5)	114 (335.6)	49 (938.8)
Vz (L)	AM	862 (224)	544 (135)	433 (120)	343 (142)	294 (238)
	GM	839 (25.3)	527 (29.1)	419 (27.0)	290 (90.8)	193 (161.3)

AM indicates arithmetic mean (SD); AUC, area under the concentration‐time curve; CL, clearance; C_max_, maximum concentration; ESRD, end‐stage renal disease; CVb, between‐subject coefficient of variation; GM, geometric mean (percentage within CVb); max, maximum; min, minimum; N, number of subjects in the treatment group; t_½_, terminal half‐life of elimination; T_max_, time from dose to C_max_; V_ss_, volume of distribution at steady state; Vz, volume of distribution in the terminal phase.

Geometric mean clearance and volume of distribution of the terminal phase (Vz) progressively decreased in subjects with increased renal impairment relative to normal renal function (Table 3). The Vz decreased with decreasing renal function and ranged from 527 L in moderate to 193 L in severe renal impairment/ESRD on hemodialysis (after hemodialysis). Terminal half‐life (t_½_) was similar between normal renal function (11.5 hours) and renal impairment (range 9.45 to 11.1 hours). In the normal‐renal‐function group, Vz was 839 L.

**Figure 1:**
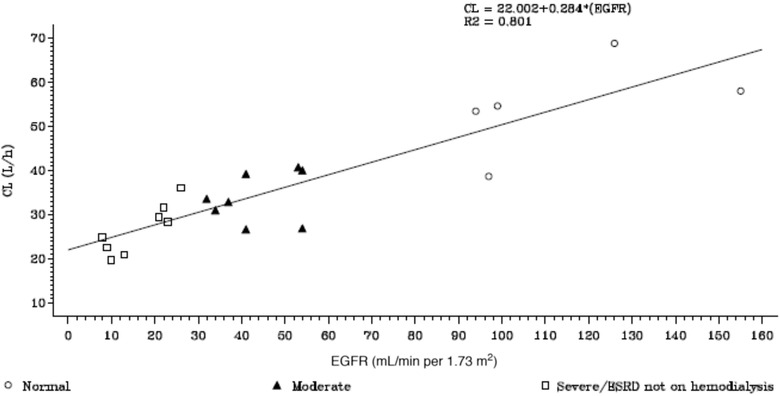
Relationship between gepotidacin clearance and renal function. CL indicates clearance; EGFR, estimated glomerular filtration rate; ESRD, end‐stage renal disease; R2, R^2^ (correlation coefficient).

There was a significant positive linear relationship (R^2 ^> 0.801; *P* < .001) of plasma CL with eGFR, where CL increased as eGFR increased (ie, renal function increased) (Figure 1).

#### PK in Urine

Urine gepotidacin exposure, renal clearance, and amount excreted were lower with decreased renal function (Table 4). Relative to normal renal function, the geometric mean urine partial AUCs (AUC_0‐12_, AUC_0‐24_, and AUC_0‐48_) were decreased more in severe/ESRD subjects not on hemodialysis than in moderate‐impairment subjects. Similarly, the renal clearance (CL_r_) was decreased by similar rates. Although CL_r_ was more impacted in ESRD subjects on hemodialysis, there were only 3 subjects who produced limited amounts of urine to measure gepotidacin. In normal‐renal‐function subjects, 37.4% of the gepotidacin dose was removed in the urine (fe%). As expected, the amount decreased to 22.1% in moderate subjects and 7.9% in ESRD subjects not on hemodialysis. Less than 2% of the gepotidacin dose was excreted in the urine for the ESRD subjects on hemodialysis.

**Table 4:** Summary of Gepotidacin Urine Pharmacokinetic Parameters by Renal Function

		Part 1			Part 2	
		Normal Renal Function	Moderate Renal Impairment	Severe/ESRD not on Hemodialysis	ESRD on Hemodialysis (Before Hemodialysis)	ESRD on Hemodialysis (After Hemodialysis)
Parameter	Units	(N = 8)	(N = 8)	(N = 8)	(N = 8)	(N = 8)
AUC_0‐12_ (μg⋅h/mL)	AM	2507 (510)	1731 (728)	555 (203)	479 (322)^b^	453 (320)^b^
	GM	2426 (20.6)	1608 (42.7)	512 (50.0)	372 (127.1)^b^	356 (117.4)^b^
AUC_0‐24_ (μg⋅h/mL)	AM	2792 (579)	1924 (738)	672 (253)	795 (138)^c^	567 (NC)^d^
	GM	2743 (20.1)	1808 (39.0)	619 (49.6)	789 (17.5)^c^	567 (NC)^d^
AUC_0‐48_ (μg⋅h/mL)	AM	2998 (649)^a^	1944 (781)^a^	736 (272)	860 (145)^c^	636 (NC)^d^
	GM	2941 (21.2)^a^	1827 (38.6)^a^	682 (47.9)	854 (17.1)^c^	636 (NC)^d^
CL_r_ (L/h)	AM	19.5 (3.70)	8.33 (4.70)	2.61 (1.66)	0.37 (0.296)^b^	0.17 (0.141)^b^
	GM	19.24 (18.8)	7.58 (44.3)	2.13 (84.3)	0.29 (101.7)^b^	0.10 (277.5)^b^
fe% (%)	AM	37.8 (5.74)	23.7 (10.6)	9.07 (4.23)	1.29 (0.938)^b^	1.64 (1.03)^b^
	GM	37.40 (15.2)	22.11 (39.4)	7.92 (68.3)	1.05 (96.4)^b^	1.45 (64.7)^b^
Ae total (mg)	AM	283 (43.1)	178 (79.3)	68.0 (31.7)	9.65 (7.03)^b^	12.3 (7.72)^b^
	GM	280.49 (15.2)	165.86 (39.4)	59.40 (68.3)	7.87 (96.4)^b^	10.87 (64.7)^b^

Ae total indicates total unchanged drug excreted; AM, arithmetic mean (SD); AUC, area under the concentration‐time curve (subscripts indicate time range); CL_r_, renal clearance; CVb, between‐subject coefficient of variation; ESRD, end‐stage renal disease; fe%, percentage of drug dose excreted in urine; GM, geometric mean (%CVb); N, number of subjects in the treatment group; NC, not calculated.

a n = 7.

b n = 3.

c n = 2.

d n = 1.

**Figure 2:**
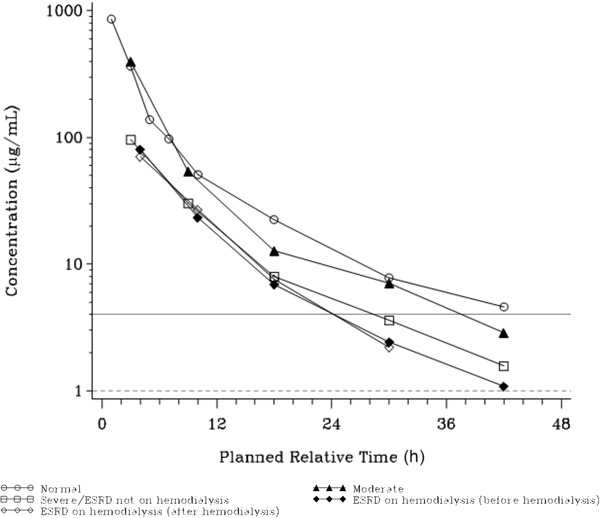
Mean gepotidacin urine concentrations by renal function (semilogarithmic scale). Lower limit of quantification (LLOQ) = 1.00 μg/mL represented by dashed line; *E coli* MIC = 4 μg/mL, represented by solid line. All values below LLOQ were set to 0. Subjects with ESRD on hemodialysis received gepotidacin 750 mg starting 2 hours before initiation of the last hemodialysis (period 1; ESRD before hemodialysis) and starting 2 hours after completion of the last hemodialysis (period 2; ESRD after hemodialysis). ESRD indicates end‐stage renal disease; MIC, minimal inhibitory concentration.

Urine concentrations generally remained measurable over the entire 48‐hour observation of the study except in ESRD subjects on hemodialysis and remained above a MIC of 4 μg/mL for more than 12 hours in all groups (Figure 2).

#### PK in Saliva

After IV administration, mean gepotidacin saliva concentrations were consistently elevated in subjects with moderate renal impairment and severe impairment/ESRD not on hemodialysis compared with normal subjects (Table 5). Saliva concentrations remained measurable over the entire 48‐hour observation of the study.

**Table 5:** Summary of Gepotidacin Saliva Pharmacokinetic Parameters by Renal Function

		Part 1		
Parameter^a^	Units	Normal Renal Function (N = 8)	Moderate Renal Impairment (N = 8)	Severe/ESRD not on Hemodialysis (N = 8)
AUC_0‐∞_ (μg⋅h/mL)	AM	9.90 (1.67)	15.2 (5.38)	17.3 (6.57)
	GM	9.78 (16.6)	14.33 (37.4)	16.33 (39.1)
C_max_ (μg/mL)	AM	2.85 (0.702)	2.71 (0.963)	3.54 (1.47)
	GM	2.76 (29.3)	2.58 (33.8)	3.26 (45.9)
T_max_ (h)	Median (min, max)	1.96 (1.42, 2.50)	1.92 (1.42, 2.00)	2.00 (1.50, 3.00)
t_½_ (h)	AM	10.1 (3.02)	10.0 (3.16)	8.85 (1.51)
	GM	9.60 (36.4)	9.71 (26.4)	8.73 (17.6)
CL (L/h)	AM	77.6 (12.6)	55.5 (20.5)	48.8 (18.0)
	GM	76.70 (16.6)	52.34 (37.4)	45.94 (39.1)
RAUC_0‐∞_ (ratio)	AM	0.987 (0.190)	1.01 (0.320)	0.914 (0.377)
	GM	0.973 (18.5)	0.956 (40.4)	0.860 (37.2)

AM indicates arithmetic mean (SD); AUC, area under the concentration‐time curve (subscripts indicate time range); CL, clearance; C_max_, maximum concentration; CVb, between‐subject coefficient of variation; ESRD, end‐stage renal disease; GM, geometric mean (%CVb); max, maximum; min, minimum; N, number of subjects in the treatment group; RAUC, the ratio of AUC observed in saliva relative to the unbound AUC in plasma; t_½_, terminal half‐life of elimination; T_max_, time from dose to C_max_.

^a^Gepotidacin PK parameters are based on unbound drug concentration in saliva (ie, no adjustment for protein binding was necessary).

Relative to normal renal function, the geometric mean C_max_ in saliva was comparable to that in subjects with moderate and severe impairment/ESRD not on hemodialysis. The geometric mean AUC_0‐t_ in saliva was elevated in all renal‐impairment subjects, but not to the extent observed in plasma. Gepotidacin saliva concentrations displayed a strong positive correlation to both total and unbound plasma concentrations (R^2^ = 0.820). When plasma parameters were corrected for protein binding (67% of fraction unbound to protein), the saliva PK parameters were comparable to the unbound plasma PK parameters.

As a result of the total exposure, CL as measured by saliva concentrations was lower in subjects with renal impairment relative to normal renal function. The t_½_ in saliva was not overly impacted in the moderate impairment and severe/ESRD not on hemodialysis, and the geometric mean t_½_ was 9.7 and 8.7 hours in moderate impairment and severe/ESRD not on hemodialysis groups, respectively, compared with 9.6 hours in the normal‐renal‐function group.

**Figure 3:**
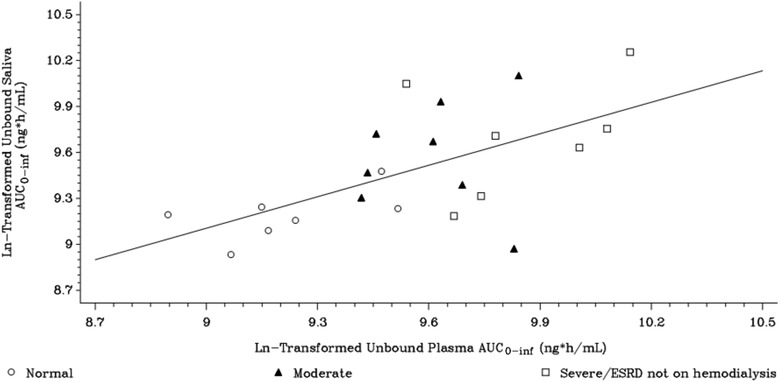
Scatterplot of gepotidacin saliva and unbound plasma AUC_0‐∞_ by renal function (log‐log plot). Plasma parameters are presented as protein‐corrected values (67% protein correction factor): unbound plasma parameters = total plasma parameter × 0.67. Saliva parameters represent unbound drug parameters because saliva drug concentrations do not require correction for protein binding. AUC indicates area under the concentration‐time curve; ESRD, end‐stage renal disease.

The ratio of unbound saliva AUC to unbound plasma AUC was approximately at unity in normal and moderate renal impairment groups (range of geometric mean ratios 0.94 to 1.0) and was slightly lower (0.85) in the severe/ESRD not on hemodialysis group. Linear regression of unbound plasma versus saliva AUC_0‐∞_ provides a slope of 0.685, which approximates the free fraction value in plasma (Figure 3).

**Figure 4:**
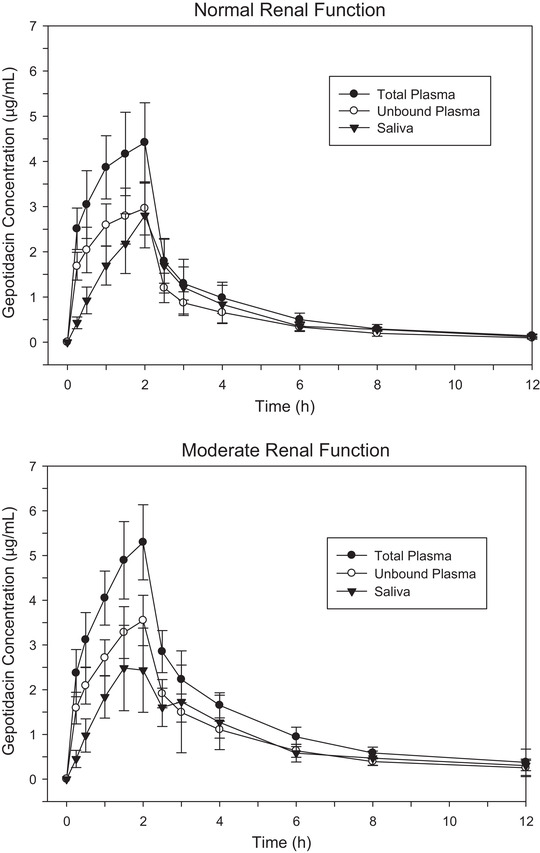
Plot of gepotidacin total plasma, unbound plasma, and saliva concentrations in normal subjects and subjects with moderate renal impairment.

Comparative plots of total plasma, unbound plasma, and saliva gepotidacin concentration‐time profiles in normal and moderate renal impairment subjects are provided in Figure 4.

#### PK in Dialysate

The amount of gepotidacin removed during dialysis decreased relative to the time on hemodialysis (Table 6). The geometric mean amount of drug removed was highest over the first hour, and the geometric mean dialysis clearance (CL_d_) over 4 hours was 6.63 L/h, comparable to the urine CL_r_ seen in moderate renal impairment subjects (geometric mean 7.58 L/h). Over a 4‐hour dialysis session, only 5.89% of the gepotidacin dose was removed by hemodialysis.

**Table 6:** Gepotidacin Dialysate Parameters Data for ESRD on Hemodialysis

		ESRD on Hemodialysis (Before Hemodialysis)
Parameter	Units	(N = 8)
Arem_0‐1_ (mg)	Arithmetic mean (SD)	18.8 (4.99)
	Geometric mean (%CVb)	18.22 (27.7)
Arem_1‐2_ (mg)	Arithmetic mean (SD)	13.1 (4.41)
	Geometric mean (%CVb)	12.42 (34.8)
Arem_2‐3_ (mg)	Arithmetic mean (SD)	9.33 (2.99)
	Geometric mean (%CVb)	8.93 (32.5)
Arem_3‐4_ (mg)	Arithmetic mean (SD)	5.63 (1.91)^a^
	Geometric mean (%CVb)	5.35 (36.6)^a^
Arem_0‐4_ (mg)	Arithmetic mean (SD)	45.4 (11.2)
	Geometric mean (%CVb)	44.15 (26.2)
AUC_0‐4_ (μg⋅h/mL)	Arithmetic mean (SD)	1.12 (0.389)^a^
	Geometric mean (%CVb)	1.06 (36.3)^a^
CL_d_ (L/h)	Arithmetic mean (SD)	6.70 (1.11)
	Geometric mean (%CVb)	6.63 (15.8)
Frem% (0‐4) (%)	Arithmetic mean (SD)	6.05 (1.50)
	Geometric mean (%CVb)	5.89 (26.2)

Arem indicates amount of drug removed (subscripts indicate time range); AUC_0‐4_, area under the concentration‐time curve over 4 hours following dose; CL, clearance; CVb, between‐subject coefficient of variation; ESRD, end‐stage renal disease; Frem, fraction of drug removed; N, number of subjects in the treatment group.

a n = 6.

### Safety

Overall, there were no AEs of clinical concern in subjects with moderate renal impairment or severe renal impairment/ESRD not on hemodialysis and in subjects with ESRD before and after hemodialysis. Subjects with normal renal function did not report any AEs. There were no AEs leading to withdrawals and no reported deaths or other serious AEs.

The majority of AEs were mild in severity and considered related to gepotidacin by the investigator. The most commonly reported AEs were gastrointestinal in nature, namely abdominal pain and diarrhea for parts 1 and 2, respectively. All AEs resolved by the end of the study. There were no clinically significant changes in vital signs, ECGs, or clinical laboratory parameters during this study.

## Discussion

This was a phase I, nonrandomized, open‐label, parallel‐group, multicenter, multipart study that evaluated the PK, safety, and tolerability of a single IV dose of gepotidacin 750 mg over 2 hours in subjects with normal renal function, subjects with moderate and severe renal impairment, and subjects with ESRD (on dialysis and not on dialysis).

Overall, administration of single 2‐hour IV doses of gepotidacin 750 mg was safe and generally tolerated with no severe AEs or withdrawal due to AEs. There were no AEs in subjects with normal renal function. In subjects with moderate and severe renal impairment and subjects with ESRD (on hemodialysis and not on hemodialysis), there were few AEs, and most were mild in intensity. The safety profile for gepotidacin in this study in subjects with different levels of renal impairment was consistent with that observed in previous studies conducted in healthy subjects.

Review of data from part 1 of the study showed that PK requirements were met (observed mean values of area under the curve [AUC_0‐∞_ < 48 μg⋅r/mL and C_max_ < 14 μg/mL]), and it was safe to proceed with part 2 of the study. Subjects with mild renal impairment were not studied because the difference in PK between subjects with moderate renal impairment and subjects with normal renal function were not considered to be clinically relevant (1.5‐fold increase in AUCs and only 1.2‐fold increase in C_max_ in the moderate‐renal‐impairment group).

Gepotidacin exposure predicted in a population with normal renal function (eGFR ≥ 90 mL/min per 1.73 m^2^) or with moderate (eGFR 30 to 59 mL/min per 1.73 m^2^) or severe renal impairment (eGFR < 30 mL/min per 1.73 m^2^) was overall consistent with observed data in the renal impairment study in which 750 mg was infused over 2 hours. Comparison of the predicted PK parameter values in Table 2 with the corresponding observed values in Table 3 confirmed that the PBPK model predictions agreed well with the observed data for all 3 groups in part 1. The predicted and observed mean values differed by 4% to 20% for AUC_0‐t_ and 12% to 37% for C_max_ across the 3 groups in part 1.

As expected, renal impairment had a direct impact on the plasma exposures and clearance of gepotidacin. A significant positive linear relationship (R^2 ^> 0.80; *P* < .001) of plasma CL with eGFR was observed. Gepotidacin dosing in subjects with severe renal impairment with and without hemodialysis resulted in significant increases in plasma drug levels and decreases in clearance. In moderate to severe nonhemodialysis ESRD subjects, C_max_ and AUC of gepotidacin increased by up to 1.6‐ and 1.9‐fold, respectively, compared with those with normal renal function. In dialysis‐dependent ESRD subjects, C_max_ and AUC of gepotidacin increased by up to 6.0‐ and 4.1‐fold, respectively, compared with those with normal renal function. Clearance values ranged from 33.53 L/h in subjects with moderate renal impairment to 12.40 L/h in hemodialysis‐dependent ESRD subjects compared with 50.55 L/h in subjects with normal renal function. The geometric mean t_½_ was minimally impacted in all the renal impairment groups and ranged from 9.45 to 11.1 hours compared with 11.5 hours in the normal‐renal‐function group. This was due to clearance, and terminal‐phase Vz progressively decreased in subjects with increase in renal impairment relative to normal renal function.

Subjects with normal renal function had 37% of the drug extracted in urine (fe%), whereas moderate‐ and severe‐renal‐impairment subjects had approximately 22% and 8% removed in urine (fe%), respectively. All ESRD subjects on hemodialysis had approximately 1.1% to 1.5% of the drug removed in urine regardless of hemodialysis timing. Although CL_r_ was more impacted in ESRD subjects on hemodialysis, there were only 3 subjects who produced limited amounts of urine to measure gepotidacin. Regardless of renal function, mean gepotidacin urine concentrations remained above an MIC of 4 μg/mL for more than 12 hours in all groups. Comparison of plasma systemic CL to CL_r_ suggests that systemic clearance primarily represents nonrenal clearance when renal clearance is severely compromised, and in these situations, nonrenal clearance plays a major role in the elimination of gepotidacin (ie, potential upregulation of other CL processes).

Comparative saliva concentrations displayed a linear relationship with plasma (both bound and unbound) gepotidacin concentrations (R^2 ^ =  0.82). The ratio of saliva AUC to unbound plasma AUC was consistently close to unity and ranged from 0.860 to 0.973. Saliva AUCs and CL displayed a linear relationship with respect to plasma AUCs and CL; the trend did not have a high correlation coefficient compared with the saliva and plasma concentration relationship, possibly due to smaller sample size. Terminal‐phase half‐life in saliva was not impacted in the moderate and ESRD subjects (8.7 to 9.7 hours compared with 9.6 hours in normal subjects) and was comparable to the respective t_½_ in plasma.

It should be noted that urine and saliva drug concentrations are considered unbound drug and did not require adjustment for protein binding.

The amount of gepotidacin removed during dialysis decreased relative to the time on hemodialysis. The CL_d_ for ESRD subjects was 6.63 L/h, which was comparable to the urine CL_r_ seen in moderate‐renal‐impairment subjects (7.58 L/h). Over a 4‐hour dialysis session, approximately 6% of the gepotidacin dose was removed by hemodialysis, possibly due to the high terminal volume of distribution of gepotidacin (geometric mean Vz of 839 L in normal renal function group), binding to plasma proteins, and significant nonrenal clearance pathways.

After IV administration, mean gepotidacin concentrations in plasma and saliva were consistently elevated in subjects with moderate renal impairment and severe/ESRD compared with normal subjects. In contrast, mean gepotidacin concentrations in urine were consistently lower in subjects with renal impairment compared with normal renal function. All mean concentrations remained measurable over the entire 48‐hour observation of the study.

In summary, administration of a single 2‐hour IV dose of gepotidacin 750 mg was safe and generally tolerated in normal and renally impaired subjects. Like most other antibiotics, gepotidacin dosing in subjects with severe renal impairment with and without hemodialysis resulted in significant increases in plasma drug levels and decreases in clearance. However, hemodialysis did not remove a significant amount of gepotidacin, which needs to be considered in case of drug overdose. Although urine drug levels decreased with decreasing renal function, the gepotidacin urine concentrations were within the target levels for efficacy, relevant for treatment of urinary tract infections. In addition, the ability to detect gepotidacin in saliva samples would allow for the use of saliva sampling to measure drug levels in populations for whom invasive blood sampling would not be feasible (eg, pediatrics).

## Funding

Funding for this study was provided by GlaxoSmithKline (NCT02729038). This work was also supported in whole or in part with federal funds from the Office of the Assistant Secretary for Preparedness and Response, Biomedical Advanced Research and Development Authority, under Other Transaction Authority Agreement No. HHSO100201300011C. Editorial support (development of the first draft, assembling tables and figures, collating author comments, and referencing) was provided by Guissou Dabiri, PhD, and was funded by GSK.

## Conflicts of Interest

M.H., C.T., A.R., D.N., G.T., and E.D. were employees of GSK and hold company stock. H.A., R.P., and T.M. were the study investigators funded by GSK during the conduct of the study. All authors meet the criteria for authorship set forth by the International Committee for Medical Journal Editors.

## Data‐Sharing Statement

Anonymized individual participant data and study documents can be requested for further research from www.clinicalstudydatarequest.com.

## Supporting information

Supporting Information.
