# Supplementary material for: Pharmacokinetics of Gepotidacin in Renal Impairment
Source: Clin Pharmacol Drug Dev. 2020 May 19;9(5):560–72. doi: 10.1002/cpdd.807 (PMC7384084; doi:10.1002/cpdd.807)
Supplement: Supplementary file 1 — Supporting Information. [file CPDD-9-560-s001.docx]

# Supplemental Data

**Supplemental Table 1 Gepotidacin SimCYP™ Simulation Parameters**

| **Simcyp Version 16** |  |  |
| --- | --- | --- |
| **Parameter** | **Values** | **Units** |
| Compound Name | GEPOTIDACIN |  |
| Infusion Dose | Various Doses | mg or mg/kg |
| Infusion Duration | 2.0 | hr |
|  |  |  |
| **PhysChem and Blood** |  |  |
| Molecular Weight | 448.5 |  |
| Log P | 0.16 |  |
| Compound Type | Diprotic Base |  |
| pKa 1 | 8.83 |  |
| pKa 2 | 6.20 |  |
| BP (Tissue: Plasma Partition) | User |  |
| Haematocrit | 45 | Simcyp Default |
| fu Input |  | User Input |
| fu | 0.67 |  |
|  |  |  |
| **Absorption** | NA | IV Route |
| **Distribution** |  |  |
| Distribution Model | Full PBPK Model |  |
| V_ss_ (Tissue: Plasma Partition Coefficients) | 1.66 | L/kg |
| Ka_AP_ (Tissue: Plasma Partition Coefficients) | 0.453 |  |
| logP_vo:w_ (Tissue: Plasma Partition Coefficients) |  |  |
| Adipose (Tissue: Plasma Partition Coefficients) | 0.487 |  |
| Bone (Tissue: Plasma Partition Coefficients) | 1.218 |  |
| Brain (Tissue: Plasma Partition Coefficients) | 1.602 |  |
| Gut (Tissue: Plasma Partition Coefficients) | 3.270 |  |
| Heart (Tissue: Plasma Partition Coefficients) | 3.473 |  |
| Kidney (Tissue: Plasma Partition Coefficients) | 3.075 |  |
| Liver (Tissue: Plasma Partition Coefficients) | 5.249 |  |
| Lung (Tissue: Plasma Partition Coefficients) | 1.466 |  |
| Muscle (Tissue: Plasma Partition Coefficients) | 3.261 |  |
| Skin (Tissue: Plasma Partition Coefficients) | 1.648 |  |
| Spleen (Tissue: Plasma Partition Coefficients) | 3.435 |  |
| Pancreas (Tissue: Plasma Partition Coefficients) | 2.614 |  |
| Addition Organ (Tissue: Plasma Partition Coefficients) with Perfusion Limited | 100 |  |
| K_p_ Scalar | 1.0 |  |
|  |  |  |
| **Elimination** |  |  |
| Clearance Type | Enzyme Kinetics |  |
| *In vitro* metabolic system | Recombinant CYPs |  |
| Enzyme (CYP3A4) Clint | 0.043 | uL/min/pmol |
| Fumic | 1.0 | Simcyp Default |
| Additional HLM Clint (uL/min/mg protein) | 9.007 | uL/min/mg protein |
| CLrenal | 16 | L/hr |

Fu= Albumin unbound fraction; Kp= Tissue:Plasma Partition Coefficient; Fu mic= Microsomal unbound fraction; Clint = Intrinsic Clearance

Supplemental Table 2

Gepotidacin Single Dose IV Pharmacokinetic Parameters (SimCYP™ Predicted Plasma vs. Observed Blood from Healthy Adult Caucasian Subjects)

| **Dose (mg)** | **Regimen** | **PK Parameter**^1^ | **Predicted^2^ (N=100)** | **Observed^3^**  **(n=6)** |
| --- | --- | --- | --- | --- |
| 400 | Single dose IV 2 hr infusion | C_max_ (μg/mL) | 2.83 (18) | 3.05 (30) |
|  |  | AUC_(0-t)_ **(**μg.hr/mL) | 9.74 (23) | 8.77 (20) |
|  |  | CL (L/hr) | 41.1 (22) | 44.5 (19) |
| 1000 | Single dose IV 2 hr infusion | C_max_ (μg/mL) | 7.10 (18) | 7.24 (26) |
|  |  | AUC_(0-t)_ (μg.hr/mL) | 24.3 (23) | 23.8 (28) |
|  |  | CL (L/hr) | 41.1 (22) | 41.6 (27) |
| 1800 | Single dose IV 2 hr infusion | C_max_ (μg/mL) | 12.7 (18) | 13.3 (14) |
|  |  | AUC_(0-t)_ (μg.hr/mL) | 43.8 (23) | 47.6 (12) |
|  |  | CL (L/hr) | 41.1 (22) | 37.6 (12) |
| ^1^Geometric mean (CV%)  ^2^ Predicted values in plasma were compared to observed values in blood since measured B/P ratio is 0.95  ^3^ Observed data for single dose Day 1  CV% =Coefficient of variation  N or n= number of virtual or enrolled subjects, respectively | | | | |

**Supplemental Table 3**

**Gepotidacin Single and Repeat Dose IV Pharmacokinetic Parameters (SimCYP™ Predicted Plasma vs. Observed Blood from Healthy Adult Caucasian Subjects)**

| **Dose (mg)** | **PK Parameter^1^** | **Day 1** | | **Day 10** | |
| --- | --- | --- | --- | --- | --- |
|  |  | **Predicted^2^ (N=100)** | **Observed^3^**  **(n=6)** | **Predicted^2^ (N=100)** | **Observed^3^**  **(n=4)** |
| 1500 | C_max_ (μg/mL) | 8.09 (18) | 7.87 (13) | 9.01 (19) | 9.30 (15) |
|  | AUC_(0-t)_ **(**μg.hr/mL) | 36.5 (23) | 34.0 (11) | 37.2^4^ (23) | 38.9^4^ (13) |
|  | CL (L/hr) | 41.1 (22) | 43.8 (11) | 40.3 (23) | 38.6 (13) |
| ^1^Geometric mean (CV%)  ^2^ Predicted values in plasma was compared to observed values in blood since measured B/P ratio is 0.95  ^3^ Observed data from single dose Day 1 and repeat TID dose on Day 10  ^4^ AUC_(0-τ)_ for Day 10 with τ = 8 hrs  N or n= number of virtual or enrolled subjects respectively | | | | | |
